# Active Recovery After High-Intensity Interval-Training Does Not Attenuate Training Adaptation

**DOI:** 10.3389/fphys.2018.00415

**Published:** 2018-04-18

**Authors:** Thimo Wiewelhove, Christoph Schneider, Alina Schmidt, Alexander Döweling, Tim Meyer, Michael Kellmann, Mark Pfeiffer, Alexander Ferrauti

**Affiliations:** ^1^Faculty of Sport Science, Ruhr-University Bochum, Bochum, Germany; ^2^Institute of Sports and Preventive Medicine, Saarland University, Saarbrücken, Germany; ^3^School of Human Movement Studies and School of Psychology, The University of Queensland, Brisbane, QLD, Australia; ^4^Institute of Sports Science, Johannes Gutenberg University, Mainz, Germany

**Keywords:** endurance training, recovery, lactate, adaptation, VO_2_max

## Abstract

**Objective:** High-intensity interval training (HIIT) can be extremely demanding and can consequently produce high blood lactate levels. Previous studies have shown that lactate is a potent metabolic stimulus, which is important for adaptation. Active recovery (ACT) after intensive exercise, however, enhances blood lactate removal in comparison with passive recovery (PAS) and, consequently, may attenuate endurance performance improvements. Therefore, the aim of this study was to examine the influence of regular ACT on training adaptations during a HIIT mesocycle.

**Methods:** Twenty-six well-trained male intermittent sport athletes (age: 23.5 ± 2.5 years; 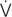
O_2_max: 55.36 ± 3.69 ml min kg^-1^) participated in a randomized controlled trial consisting of 4 weeks of a running-based HIIT mesocycle with a total of 12 HIIT sessions. After each training session, participants completed 15 min of either moderate jogging (ACT) or PAS. Subjects were matched to the ACT or PAS groups according to age and performance. Before the HIIT program and 1 week after the last training session, the athletes performed a progressive incremental exercise test on a motor-driven treadmill to determine 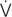
O_2_max, maximum running velocity (vmax), the running velocity at which 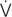
O_2_max occurs (v
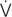
O_2_max), and anaerobic lactate threshold (AT). Furthermore, repeated sprint ability (RSA) were determined.

**Results:** In the whole group the HIIT mesocycle induced significant or small to moderate changes in vmax (*p* < 0.001, effect size [ES] = 0.65,), v
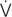
O_2_max (*p* < 0.001, ES = 0.62), and AT (*p* < 0.001, ES = 0.56) compared with the values before the intervention. 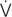
O_2_max and RSA remained unchanged throughout the study. In addition, no significant differences in the changes were noted in any of the parameters between ACT and PAS except for AT (*p* < 0.05, ES = 0.57).

**Conclusion:** Regular use of individualized ACT did not attenuate training adaptations during a HIIT mesocycle compared to PAS. Interestingly, we found that the ACT group obtained a significantly higher AT following the training program compared to the PAS group. This could be because ACT allows a continuation of the training at a low intensity and may activate specific adaptive mechanisms that are not triggered during PAS.

## Introduction

Various high-intensity interval training (HIIT) protocols are frequently used in modern training programs and especially in intermittent sports, such as team or racket sports, to enhance the fitness of the athletes (Iaia et al., 2009). The physiological adaptations associated with HIIT are based on complex molecular and cellular mechanisms in response to different metabolic and mechanical stimuli (Wahl et al., 2010a). In this context, lactate may play an important role as it can initiate specific adaptive processes. With regard to acute physiological responses, HIIT protocols (especially those with long intervals at intensities near the power output at 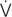
O_2_max) typically lead to extremely high blood lactate levels (Tschakert and Hofmann, 2013; Wiewelhove et al., 2016a). This transient increase in circulating lactate in turn induces oxygen radical generation, which activates a transcriptional network signaling adaptive cell responses (e.g., angiogenesis, vasculogenesis, and mitochondrial biogenesis; Brooks et al., 2008). Many adaptations to HIIT may therefore be mediated in some way by lactate.

Immediately after a fatiguing exercise such as HIIT, active recovery (ACT) as part of the training during the cooldown phase is commonly used in daily practice among athletes with the aim of preserving performance levels between training sessions, especially if athletes complete more than one session per day (Barnett, 2006; Le Meur and Hausswirth, 2013; Kellmann et al., 2018). ACT strategies usually consist of aerobic-type, whole-body activities (e.g., running, biking, or swimming), performed at loads between 30 and 60% of the individual’s maximal oxygen consumption (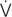
O_2_max) and lasting for at least 15 min (Nédélec et al., 2013; Wiewelhove et al., 2016b). From a short-term perspective, there is inconsistent and limited evidence that ACT actually enhances recovery between events. However, most studies have unanimously demonstrated that ACT allows a faster return to resting lactate levels than passive recovery (PAS; Barnett, 2006). This is because maintaining submaximal activity after intense exercise, leading to a significant increase in circulating lactate, effectively favors oxidation of the lactic acid, mainly by the active muscle fibers (Le Meur and Hausswirth, 2013).

Consequently, ACT completed immediately after HIIT might lead to shorter metabolic alterations. However, from a long-term perspective, the extent of metabolic stress is potentially important for the physiological adaptations and performance improvements associated with HIIT as described above. In order to increase the efficiency of HIIT, the arrangement of post-training recovery is therefore possibly just as important as the prescription of the training itself. In this context, a study by Wahl et al. (2013) demonstrated that PAS during a HIIT shock microcycle allowed athletes to improve endurance performance to a greater extent than with ACT. However, in this study, the different recovery strategies were applied only during training (i.e., between intervals), while we were unable to find any studies that have examined the influence of regular and extensive use of post-exercise ACT on training adaptations.

As such, the purpose of the current study was (1) to investigate the effect of a HIIT mesocycle during a preparation period (i.e., the off-season) on physiological adaptations and endurance performance of team and racket sport athletes and (2) to assess whether the arrangement of the post-training recovery (ACT versus PAS) would elicit different results. We hypothesized (1) that the 4-week training program, including 12 HIIT sessions among other things, leads to an improvement in 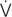
O_2_max, anaerobic lactate threshold, and maximum running velocity in a progressive incremental exercise test and (2) that the regular use of ACT immediately after each HIIT session attenuates the expected training adaptations.

## Materials and Methods

### Participants

Thirty healthy and well-trained male intermittent sport athletes (i.e., soccer, handball, basketball, hockey, floorball, tennis) were recruited to take part in this study. Four athletes dropped out due to injuries that were not related to the study intervention. The baseline physical characteristics of the remaining 26 athletes whose data were included in the data analysis are shown in **Table 1**. After being informed about the exercise protocols and all possible risks associated with participation in the investigation, the players provided written consent to participate in all procedures. To ensure that there were no contraindications for vigorous exercise, all subjects completed the Physical Activity Readiness Questionnaire (PAR-Q; Thomas et al., 1992) before any testing took place. Additionally, athletes had to meet the following inclusion criteria: a minimal 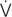
O_2_max of at least 50.00 ml min kg^-1^. The research was approved by the ethics committee of the medical faculty of the Ruhr-University Bochum (registration number: 2015-5265) and was conducted according to the guidelines of the Declaration of Helsinki.

**Table 1:** Baseline physical characteristics of the athletes.

	Age (years)	Height (cm)	Body mass (kg)	Body fat (%)	VO_2_max (ml⋅min⋅kg^-1^)	AT (km⋅h^-1^)
Overall (*n* = 26)	23.5 ± 2.5	182.8 ± 6.4	77.7 ± 6.2	12.7 ± 4.2	55.4 ± 3.7	12.5 ± 1.0
Active recovery group (*n* = 13)	24.0 ± 2.7	181.0 ± 4.1	75.8 ± 5.0	12.4 ± 3.8	55.2 ± 3.3	12.5 ± 1.1
Passive recovery group (*n* = 13)	23.0 ± 2.2	184.6 ± 7.6	79.7 ± 6.7	13.0 ± 4.6	55.5 ± 4.0	12.4 ± 0.8

*Data are shown as mean ± SD. AT, anaerobic threshold.*

### Experimental Design

**FIGURE 1:**
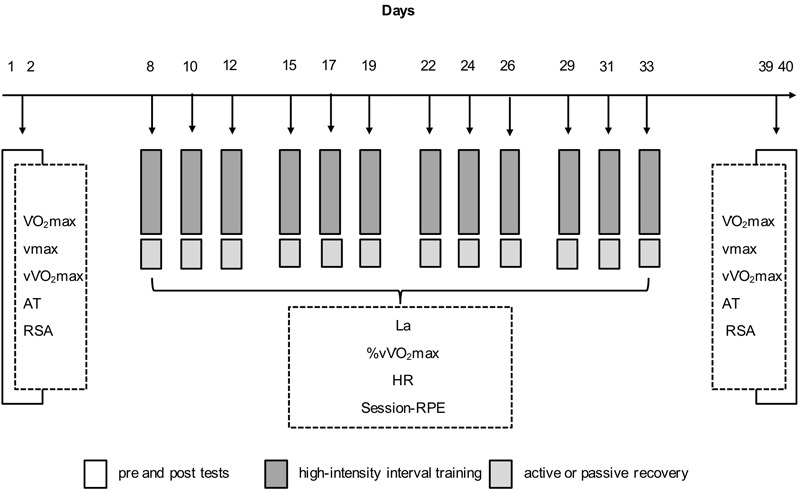
Experimental protocol showing the measurements [maximum running velocity (vmax); running velocity at which maximal oxygen consumption occurs (v
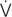
O_2_max); anaerobic lactate threshold (AT); repeated sprint ability (RSA); blood lactate concentration (La); heart rate (HR); session-rating of perceived exertion (session-RPE)] and the arrangement of the 12 high-intensity interval training (HIIT) sessions during the 4-day training period.

A matched pairs parallel design was used to investigate the influence of regular ACT on training adaptations during a HIIT mesocycle. The athletes participated in a 4-week training period, which consisted of 12 running-based HIIT sessions and was conducted during the athletes’ preparatory period (i.e., the off-season). Before the HIIT program and 1 week after the last training session (to allow for a complete recovery and supercompensation), the athletes’ 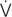
O_2_max, maximum running velocity (vmax), the running velocity at which 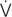
O_2_max occurs (v
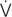
O_2_max), and anaerobic lactate threshold (AT) were determined. Furthermore, repeated-sprint abilities (RSA) and anthropometrical characteristics were measured. During the mesocycle and immediately after each training session, participants completed 15 min of either moderate jogging (ACT) or PAS in a seated position (**Figure 1**). For the assignment to ACT or PAS groups, subjects were matched into homogeneous pairs, based on age and performance. Then, within each pair, athletes were randomly assigned to one of the two experimental groups (**Table 1**).

Athletes were instructed to arrive at the testing sessions in a rested and hydrated state after fasting for at least 2 h and were told to avoid strenuous exercise for 72 h before all testing. Each subject was examined at approximately the same time of day and performance tests (conducted in the following order: incremental treadmill test, 2-h rest, RSA test) were always conducted on the same treadmills. The incremental treadmill test was performed before the RSA test and therefore in a fully recovered state because the parameters determined in the incremental treadmill test were the main depended variables of interest. To minimize possible learning effects, the RSA test was thoroughly familiarized 1 week before the first testing session. Participants were also asked to maintain their normal dietary intake and to refrain from nutritional supplements and alcohol intake during the experimental period. In this regard, athletes were verbally questioned regularly to ensure that they had adhered to the dietary rules. The verbal feedback of the athletes suggests that compliance with nutritional rules was good. Throughout the duration of the study, all subjects were required to keep a detailed training diary containing information on all training performed over that time. In addition, the Short Recovery and Stress Scale (SRSS) (Kellmann et al., 2016) was used to assess the athletes’ perceived recovery and stress state pre- and post-HIIT session.

### Procedures

#### Incremental Treadmill Test

In order to determine 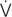
O_2_max, vmax, v
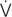
O_2_max, and AT, a progressive, incremental exercise test on a motor-driven treadmill (Saturn 300/100 r, h/p/cosmos sports and medical GmbH, Nussdorf-Traunstein, Germany; Ergo ELG2, Woodway GmbH, Weil am Rhein, Germany) was used. The protocol, which was modified according to Scharhag-Rosenberger et al. (2012), started with four submaximal exercise stages, beginning at 8 km h^-1^ and increasing by 2 km h^-1^ every 3 min with a constant incline of 0.5%. After these four stages, the test continued with ramp-wise increments of 0.8 km h^-1^ every 1 min until voluntary exhaustion. The protocol allowed for the assessment of AT, 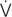
O_2_max, vmax, and v
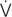
O_2_max in the same test.


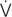
O_2_ was continuously analyzed using a breath-by-breath gas collection system (MasterScreen CPX, VIASYS Healthcare GmbH, Höchberg, Germany). The gas and volume calibrations were carried out before each test according to the instructions provided by the manufacturer. All respiratory data were averaged every 5 s, and the highest 30 s rolling average during the ramp-wise part of the test was defined as the 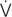
O_2_max. The v
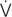
O_2_max was defined as the linear interpolated running speed when the 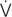
O_2_max was reached and vmax as the linear interpolated running speed at the time point of exhaustion. The v
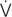
O_2_max was used to calculate the interval intensities of the HIIT protocols.

Capillary blood samples were taken from a hyperemic earlobe before the test, during a 30 s break immediately following each submaximal velocity level, and at the time of exhaustion. These were analyzed for blood lactate (La) concentration. Blood samples were taken with 20 μL capillaries, hemolyzed in 1 mL micro-test tubes and underwent amperometric-enzymatic analysis using the Biosen S-Line Sport (EKF-diagnostic GmbH, Barleben, Germany). From the resulting La values, ATs were determined according to Dickhuth et al. (1999). Heart rates (HRs) were monitored and recorded at 1 s intervals during the test (V800, Polar Electro GmbH, Büttelborn, Germany).

#### Repeated Sprint Ability Test

In order to measure RSA, a laboratory repeated-sprint test was performed on a non-motorized treadmill (NMT; Force 3.0, Woodway GmbH, Weil am Rhein, Germany). The experimental setup of the test has previously been described by Oliver et al. (2006). The RSA test consisted of 6 × 4 s maximal sprints from a standing position with a 20 s passive recovery between sprints. The peak values attained in each sprint for velocity were recorded, and the mean peak values for velocity (MV) as well as the percentage decrement score [Sdec = (1 - (peak velocity of sprint 1 + peak velocity of sprint 2 +... + peak velocity of the final sprint)/(peak velocity of the best sprint × number of sprints)) × 100; Girard et al., 2011] were calculated. For MV and Sdec, the intraclass correlation coefficient (ICC), the typical error (TE), and the coefficient of variation (CV) were previously investigated by our research group (Wiewelhove et al., 2015). MV was considered to be highly reliably [MV (m⋅s^-1^), *n* = 17, ICC = 0.92, TE = 0.10, CV = 1.5%], while Sdec was much less reliable [unpublished results: Sdec (%), *n* = 17, ICC = 0.36, TE = 1.11, CV = 34%]. This is consistent with the results of Oliver et al. (2006).

#### Perceived Recovery and Stress

Athletes’ perceived recovery and stress states were assessed using the SRSS (Kellmann et al., 2016). Subjects were requested to provide responses to eight items on a 0 (does not apply to all) to 6 (fully applies) rating scale. Numbers 1–5 on this scale were undefined and were used to delineate the degrees of perceived recovery and stress between the two ends of the scale. The items used in this study were overall recovery (OR) and overall stress (OS; Kellmann et al., 2016).

### Training Program

During the 4-week mesocycle, the athletes trained three times per week (Mondays, Wednesdays, and Fridays at approximately the same time), completing a total of 12 HIIT sessions with an average training duration of 40 min per session, which included the warm-up as well as the actual interval training protocol. To break the monotony, at each HIIT session subjects completed either 2, 3, or 4 min intervals at 100, 95, or 90% of the individual v
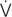
O_2_max taken from the incremental exercise test with 2, 2.5, or 3 min passive recovery periods between intervals (**Table 2**). Long-interval HIIT (i.e., an interval duration of ≥60 s and a work/rest ratio of ≥1) was chosen as it has been demonstrated to be a powerful stimulus for producing high levels of lactic acid in the blood (Buchheit and Laursen, 2013; Tschakert and Hofmann, 2013). To produce a progressive increase of training stress (i.e., volume), the number of intervals was increased for the first 3 weeks of the mesocycle, followed by a reduced number of intervals during the 4rth week of the cycle to allow for adequate recovery, restoration, and adaptation from the three buildup weeks. This resulted in the 23, 27, 31, 19% periodization recommended by Sleamaker and Browning (1996) for a 4-week training cycle.

**Table 2:** High-intensity interval training protocol characteristics.

Interval and recovery duration (min)	Interval intensity (%v 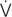 O_2_max)	Recovery intensity	Exercise mode	Number of intervals in week 1	Number of intervals in week 2	Number of intervals in week 3	Number of intervals in week 4
2,0/2,0	100	Passive	Straight-line runs	7	8	9	6
3,0/2,5	95	Passive	Straight-line runs	6	7	8	5
4,0/3,0	90	Passive	Straight-line runs	4	5	6	3

All HIIT sessions were completed outdoors on a 400 m tartan track and preceded by a standardized 10 min warm-up. To ensure that the intended training intensity was maintained by the athletes, all sessions were supervised by the same research fellows, and individually calculated running speeds, the resulting running distances, as well as the running velocities in relation to the v
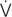
O_2_max were controlled and calculated. Additionally, HRs were monitored and recorded at 1 s intervals during each HIIT session. From the data, the training impulse (TRIMP) was calculated according to the summated heart rate zones method using the following formula: TRIMP = (duration in zone 1 × 1) + (duration in zone 2 × 2) + (duration in zone 3 × 3) + (duration in zone 4 × 4) + (duration in zone 5 × 5), where zone 1 stands for 50 to 60% of the maximum HR (HRmax), zone 2 for 60 to 70% of HRmax, zone 3 for 70 to 80% of HRmax, zone 4 for 80 to 90% of HRmax, and zone 5 for 90 to 100% of HRmax (Borresen and Lambert, 2008). Furthermore, capillary blood samples were obtained from the hyperemic earlobe throughout each training session (i.e., before the training session, immediately after the last interval, as well as during and/or immediately at the end of the recovery intervention) and analyzed for La concentrations. Blood samples were taken with 20 μL capillaries, hemolyzed in 1 mL micro-test tubes and underwent amperometric-enzymatic analysis using the Biosen S-Line Sport (EKF-diagnostic GmbH, Barleben, Germany). Moreover, the internal training load for each HIIT session were determined by using the session rating of perceived exertion (session-RPE) method (Foster et al., 2001). This method involved multiplying the absolute training duration in minutes by the training intensity. The intensity was rated by the subjects 30 min following each training session using a 10-point category-ratio RPE scale (Alexiou and Coutts, 2008). Finally, athletes used training diaries to document any other training that was completed supplementary to the HIIT sessions. The type of training, duration, and session-RPE were recorded in the training diaries for each training session completed. We could then assess the overall internal training load during the mesocycle.

### Recovery Intervention

ACT included 15 min of moderate jogging that was started within 5 min after completing each training session. The running intensity was self-paced by using the RPE method. Maximum La clearance rates during ACT after an intense running bout occur at 80–100% of AT (Menzies et al., 2010). The intensity selected was therefore 12–13 on Borg’s 6–20 RPE scale (Borg, 1998), which is equivalent to an exercise intensity close to the AT (Scherr et al., 2013). To monitor the intensity of ACT, jogging distances were recorded and the resulting running speeds as well as the intensities related to the v
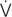
O_2_max were calculated.

### Statistical Analysis

Statistical analysis was conducted with IBM SPSS Statistics (version 23, International Business Machines Corporation, Armonk, NY, United States) and with a published spreadsheet (Hopkins, 2006). Descriptive statistics of the data are presented as mean ± SD. Furthermore, 95% confidence intervals (CIs) are given. The data were tested for normal distributions using the Shapiro–Wilk test. In cases of non-normal distributions, data were log-transformed before statistical analysis to improve normality and variance homogeneity. To check the first hypothesis that the 4-week HIIT program elicits an improvement in performance and physiological measures, pre- and post-test values of the whole group as well as of the ACT and PAS groups were compared separately using a paired *t*-test. To verify the second hypothesis that the use of regular ACT after each training session attenuates training adaptations, the absolute changes of both groups were compared with an unpaired *t*-test. Statistical significance was set at *p* < 0.05.

Furthermore, the magnitude of the changes between testing days as well as the magnitude of differences in changes between ACT and PAS was assessed using sample size-adjusted effect size (ES). The threshold values for ES were 0.2 (small), 0.6 (moderate), 1.2 (large), and 2.0 (very large; Hopkins et al., 2009). In addition, 95% CIs for the between-groups differences in changes were estimated, and magnitude-based inferences were made with reference to the smallest worthwhile change, which was calculated as 0.2 multiplied by the pooled between-subject variation of the pretests. The quantitative chances of having a harmful, trivial, or beneficial effect of ACT were assessed qualitatively as follows: <0.5%, almost certainly not; 0.5 to 5%, very unlikely; 5 to 25%, unlikely; 25 to 75%, possibly; 75 to 95%, likely; 95 to 99.5%, very likely; and >99.5%, almost certainly (Hopkins et al., 2009). If the chance of a harmful and a beneficial effect were both >2.5%, the true difference was considered to be unclear.

## Results


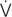
O_2_max relative to body mass and RSA (i.e., MV and Sdec) showed no significant or meaningful changes from pre to post, neither in the whole group nor in the ACT or PAS group. Vmax increased significantly or moderately over time, both in the whole group and in the ACT and PAS groups. V
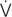
O_2_max also increased significantly in both the whole group, as well as in the ACT and PAS groups, while the change in the whole group and the ACT group was moderate and small in the PAS group. AT increased significantly only in the whole group and the ACT group, while the change was small in the whole group, moderate in the ACT group, and trivial in the PAS group. Furthermore, no significant differences in the changes were noted in any of the parameters between the ACT and PAS groups, except for AT, where a significant difference in the change was observed. In this context, magnitude-based inferences also showed a likely beneficial impact on AT for ACT compared with PAS, while the effects on 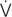
O_2_max, vmax, v
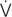
O_2_max, and RSA were neither harmful nor beneficial (**Tables 3, 4**).

**Table 3:** Maximal oxygen consumption (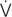
O_2_max), maximum running velocity, running velocity at which 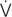
O_2_max occurs, and anaerobic lactate threshold before and after a 4-week high-intensity interval training period; the pre to post changes in mean values; and the differences in the changes between the active recovery (*n* = 13) and passive recovery (*n* = 13) group.

Variable	Group	Pre		Post		Pre to post changes				Differences in the changes between ACT and PAS				
		Mean ±*SD* (95% CI)		Mean ±*SD* (95% CI)		ΔMean ±*SD* (95% CI)		*p*	ES	ΔMean (95% CI)	*p*	ES	Qualitative inference and likelihood (%) of ACT being positive/trivial/negative	
*Treadmill test*													
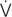 O_2_max (ml⋅min⋅kg^-1^)	Overall	55.36 ± 3.76	(53.84; 56.88)	55.28 ± 3.51	(53.86; 56.69)	-0.09 ± 2.42	(-1.07; 0.89)	0.853	-0.02					
	ACT	55.19 ± 3.45	(53.10; 57.28)	55.55 ± 3.44	(53.47; 57.63)	0.36 ± 2.43	(-1.82; 1.11)	0.603	0.09	0.90 (-1.07; 2.87)	0.355	0.21	Unclear	51/45/4
	PAS	55.54 ± 4.18	(53.01; 58.06)	55.00 ± 3.69	(52.77; 57.23)	-0.54 ± 2.43	(-0.93; 2.01)	0.440	-0.13					
vmax (km⋅h^-1^)	Overall	17.2 ± 1.0	(16.8; 17.6)	17.9 ± 0.9	(17.5; 18.2)	0.7 ± 0.4	(0.5; 0.9)	<0.001	0.65					
	ACT	17.2 ± 1.0	(16.6; 17.8)	17.8 ± 0.9	(17.3; 18.4)	0.7 ± 0.4	(0.4; 0.9)	<0.001	0.62	0.0 (-0.4; 0.3)	0.790	-0.04	Unclear	7/76/17
	PAS	17.2 ± 1.0	(16.6; 17.8)	17.9 ± 0.9	(17.3; 18.4)	0.7 ± 0.4	(0.4; 1.0)	<0.001	0.67					
v 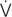 O_2_max (km⋅h^-1^)	Overall	17.0 ± 1.1	(16.6; 17.5)	17.8 ± 1.0	(17.4; 18.1)	0.7 ± 0.7	(0.5; 1.0)	<0.001	0.62					
	ACT	17.0 ± 1.2	(16.3; 17.7)	17.9 ± 1.0	(17.2; 18.5)	0.9 ± 0.7	(0.4; 1.3)	<0.01	0.73	0.2 (-0.3; 0.8)	0.349	0.19	Unclear	49/48/4
	PAS	17.0 ± 1.1	(16.3; 17.7)	17.6 ± 0.9	(17.1; 18.2)	0.6 ± 0.6	(0.3; 1.0)	<0.01	0.52					
AT (km⋅h^-1^)	Overall	12.5 ± 1.0	(12.1; 12.8)	13.0 ± 1.2	(12.6; 13.5)	0.6 ± 0.7	(0.3; 0.8)	<0.001	0.56					
	ACT	12.4 ± 0.9	(11.9; 12.9)	13.3 ± 0.8	(12.8; 13.8)	0.9 ± 0.4	(0.6; 1.2)	<0.001	0.84	0.6 (0.1; 1,1)	<0.05	0.57	Likely positive	95/5/0
	PAS	12.5 ± 1.1	(11.8; 13.2)	12.8 ± 1.4	(11.9; 13.7)	0.3 ± 0.7	(-0.1; 0.7)	0.167	0.27					

*CIs, confidence limits; ES, effect size; vmax, maximum running velocity; v
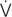
O_*2*_max, running velocity at which 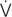
O_*2*_max occurs; AT, anaerobic lactate threshold; ACT, active recovery; PAS, passive recovery.*

**Table 4:** Repeated sprint ability before and after a 4-week high-intensity interval training period; the pre to post changes in mean values; and the differences in the changes between the active recovery (*n* = 13) and passive recovery (*n* = 13) group.

Variable	Group	Pre		Post		Pre to post changes				Differences in the changes between ACT and PAS				
		Mean ± *SD* (95% CI)		Mean ± *SD* (95% CI)		ΔMean ± *SD* (95% CI)		*p*	ES	ΔMean (95% CI)	*p*	ES	Qualitative inference and likelihood (%) of ACT being positive/trivial/negative	
*RSA test*														
MV (m⋅s^-1^)	Overall	5.61 ± 0.28	(5.49; 5.73)	5.58 ± 0.30	(5.45; 5.70)	-0.03 ± 0.12	(-0.09; 0.02)	0.173	-0.11					
	ACT	5.59 ± 0.32	(5.40; 5.78)	5.54 ± 0.33	(5.34; 5.74)	-0.05 ± 0.14	(-0.13; 0.04)	0.249	-0.16	-0.03 (-0.13; 0.08)	0.589	-0.09	Unclear	5/70/25
	PAS	5.63 ± 0.24	(5.47; 5.78)	5.61 ± 0.26	(5.44; 5.78)	-0.02 ± 0.10	(-0.09; 0.05)	0.508	-0.07					
Sdec (%)	Overall	3.45 ± 1.14	(2.98; 3.92)	3.05 ± 1.23	(2.49; 3.51)	-0.45 ± 1.35	(-1.01; 0.11)	0.108	-0.37					
	ACT	3.56 ± 0.88	(3.02; 4.09)	3.10 ± 1.31	(2.31; 3.90)	-0.45 ± 1.29	(-1.23; 0.33)	0.230	-0.37	0.00 (-1.13; 1.06)	0.949	0.00	Unclear	33/33/33
	PAS	3.34 ± 1.39	(2.46; 4.22)	3.00 ± 1.20	(2.14; 3.64)	-0.45 ± 1.48	(-1.39; 0.49)	0.313	-0.37					

*CIs, confidence limits; ES, effect size; RSA, repeated sprint ability; MV, mean peak values for velocity; Sdec, percentage decrement score; ACT, active recovery; PAS, passive recovery.*

**FIGURE 2:**
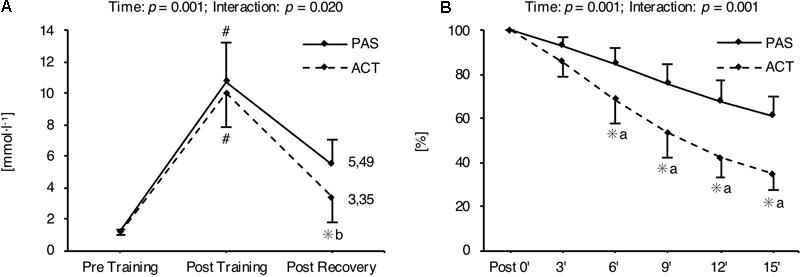
Mean (± SD) blood lactate concentration throughout each HIIT session determined before (Pre Training) and after training (Post Training), as well as after recovery (Post Recovery) **(A)**. Mean (± SD) percentage blood lactate concentration relative to post-training concentration, measured 3, 6, 9, 12, and 15 min after the 10th HIIT session **(B)**. #Significant difference compared with Pre Training and Post Recovery (*p* < 0.05); ^∗^significant difference compared with passive recovery (*p* < 0.05); ^a^moderate effect size; ^b^large effect size. ACT, active recovery; PAS, passive recovery.

The completed running distances as well as the running speeds relative to the v
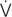
O_2_max during the 2, 3, and 4 min interval training sessions were not different between groups. The same applies to the athletes’ ratings of the difficulty of the sessions, which ranged from 7 to 8 (i.e., very hard; **Table 5**) and reflect the high intensity of the training program. Furthermore, a significant main effect for time as well as a significant recovery intervention × time interaction was found for mean La concentrations throughout each HIIT session. In both intervention groups, La concentration was significantly increased immediately after training (*p* = 0.001), and significantly decreased immediately after the recovery intervention (*p* = 0.001). La values at rest before HIIT (*p* = 0.501) as well as immediately at the end of the training (*p* = 0.377) were not different between ACT and PAS. However, La concentrations measured during (after 6, 9, and 12 min) as well as immediately at the end of the recovery intervention were significantly lower for ACT as compared with PAS (**Figure 2**).

**Table 5:** Completed running distances, mean running speeds in relation to the velocities at which maximal oxygen consumption occurs (%v
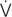
O_2_max), and session-rating of perceived exertion (session-RPE) during the 2, 3, and 4 min interval training sessions; differences in completed running distances, %v
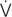
O_2_max, and session-RPE between the active recovery (*n* = 13) and passive recovery (*n* = 13) group; %v
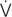
O_2_max and mean absolute running speeds during active recovery following the 2, 3, and 4 min interval training sessions.

Variable	Group	2 min intervals			3 min intervals			4 min intervals		
		Mean ± *SD*	*p*	ES	Mean ± *SD*	*p*	ES	Mean ± *SD*	*p*	ES
*High-intensity interval training*										
Completed running distances (m)	ACT	4133 ± 604	0.904	0.02	5082 ± 826	0.501	0.12	4533 ± 1034	0.974	0.01
	PAS	4120 ± 564			4985 ± 817			4528 ± 988		
%v 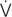 O_2_max	ACT	97.9 ± 2.5	0.635	0.18	91.4 ± 2.1	0.372	0.50	90.8 ± 2.2	0.233	0.39
	PAS	97.5 ± 2.3			92.7 ± 2.4			90.0 ± 2.7		
Session-RPE	ACT	7.7 ± 0.9	0.827	0.09	7.8 ± 0.9	0.515	0.35	6.6 ± 0.8	0.458	0.36
	PAS	7.8 ± 0.9			8.1 ± 0.8			7.0 ± 1.2		
*Active recovery*										
%v 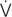 O_2_max	ACT	51.6 ± 4.3			53.3 ± 5.5			53.1 ± 5.2		
km⋅h^-1^	ACT	8.7 ± 0.8			9.0 ± 0.9			9.0 ± 0.9		

*ES, effect size; v
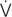
O_*2*_max, running velocity at which 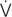
O_*2*_max occurs; Session-RPE, session-rating of perceived exertion; ACT, active recovery; PAS, passive recovery.*

**FIGURE 3:**
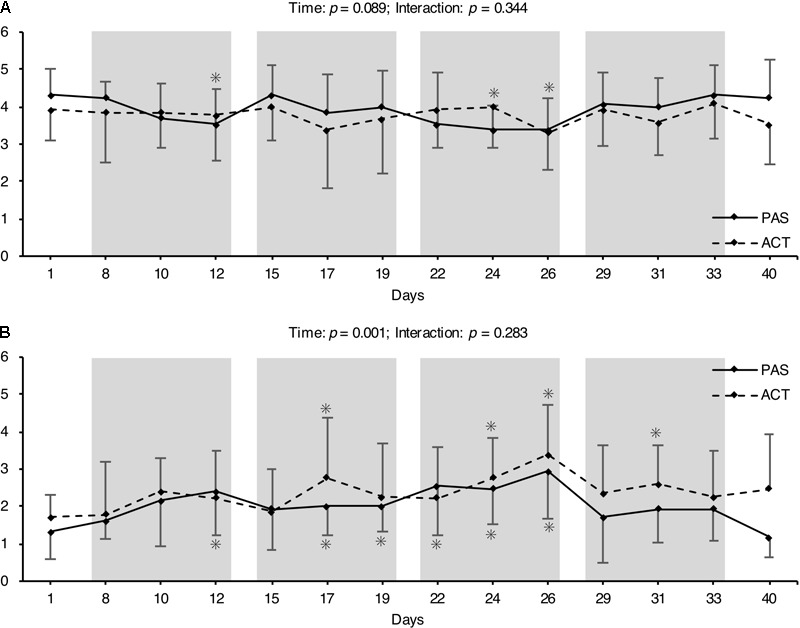
Mean (± SD) of overall recovery **(A)** and overall stress **(B)** before, during, and after the 4-week HIIT period. The gray columns mark the individual training weeks. ^∗^Significant difference compared to pre (*p* < 0.05).

Overall TRIMP (ACT: arbitrary unit [AU] = 1242 ± 103; PAS: AU = 1257 ± 152; *p* = 0.811) as well as total training load calculated using the session-RPE method (ACT: AU = 17897 ± 5428; PAS: AU = 15201 ± 3907; *p* = 0.175) also did not differ between groups. However, there was a moderate ES (i.e., 0.62) for the difference in session-RPE AU between ACT and PAS. The exercise intensities for the active recovery intervention when expressed as percentages of v
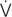
O_2_max were all within a 51–54% v
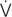
O_2_max range. This corresponded to a mean running speed of 8.7 to 9.0 km h^-1^ (**Table 5**). OS significantly increased over time, while there were no significant recovery intervention × time interactions for the perceived recovery and stress states during the experimental period (**Figure 3**).

## Discussion

To the authors’ knowledge, this is the first trial that examined the influence of the regular use of ACT immediately after HIIT on chronic physiological and performance responses. The major findings of this investigation were that a 4-week HIIT program with a total of 12 training sessions is able to improve vmax during a progressive incremental exercise test as well as v
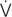
O_2_max and AT. This confirms the findings of other research studies that have demonstrated that a HIIT program performed during 4 weeks is an effective short-term strategy to alter some variables related to endurance performance in well-trained athletes (Laursen et al., 2005; Driller et al., 2009; Lamberts et al., 2009; Swart et al., 2009; Silva et al., 2017). Furthermore, the collected data showed that ACT did not attenuate training adaptations during the mesocycle compared to PAS. Interestingly, we found that the ACT group even obtained a significantly higher AT following the training program compared to the PAS group. As such, this study gives the first hint that maintaining low-intensity work during the cooldown phase is actually a means to continue training, thereby activating specific adaptive mechanisms, rather than a means to truly accelerate the recovery process. This is supported by the total training load calculated using the session-RPE method, which was 15% higher in the ACT group (ES = 0.62) compared with the PAS group because of the additional 15 min work after each HIIT session.

Besides the effect of the HIIT mesocycle on physiological adaptations and endurance performance, the present results show that the HIIT protocols were extremely demanding and produced high La levels of up to 10.8 mmol l^-1^. In this regard, the decrease in the accumulated La concentration after HIIT was accelerated when the training was followed by ACT (i.e., moderate jogging at an average speed of about 9 km h^-1^, corresponding to approximately 53% of the v
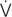
O_2_max; **Table 5**) rather than PAS. This effect is well-established and was the basis for our second hypothesis (i.e., that the regular use of ACT immediately after each HIIT session would attenuate the training adaptations) as it was shown before that La may be a potent metabolic stimulus for adaptations to training (Nalbandian and Takeda, 2016). In addition, the training program resulted in an accumulation of OS during the 4-week mesocycle. This is in line with several studies that have demonstrated the exhaustive nature of short-term intensified training cycles (Billat et al., 1999; Halson et al., 2002; Wiewelhove et al., 2015, 2017a). Interestingly, there was no difference in the increase of OS over time between intervention groups (**Figure 3**), which is also in agreement with previous research (Wiewelhove et al., 2016b) and emphasizes that ACT seems to be neither beneficial nor detrimental to the recovery process.

In accordance with the results of Dupont et al. (2004) and Fernandez-Fernandez et al. (2012), this study underlines the effectiveness of HIIT in improving endurance performance in well-trained intermittent sport athletes (i.e., team and racket sports; Dupont et al., 2004; Fernandez-Fernandez et al., 2012). Although we were not able to induce improvements of 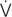
O_2_max like in the studies of Ferrari Bravo et al. (2008), Driller et al. (2009), and Fernandez-Fernandez et al. (2012), vmax, v
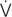
O_2_max, and AT significantly increased in the whole group by 4.1, 4.7, and 4.0%, respectively. Lamberts et al. (2009), Swart et al. (2009), and Wahl et al. (2013) also showed that a short-term HIIT program can improve endurance performance without the detection of concurrent increases in 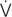
O_2_max. This might be due to the fact that the determination of 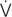
O_2_max is accompanied by methodological problems that lead to measurement inaccuracies of up to 5% (Meyer and Kindermann, 1999). Together with the large ranges in individual 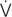
O_2_max response following training programs based on %
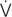
O_2_max (Mann et al., 2013), it may therefore be difficult to measure any biologically meaningful changes in 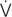
O_2_max after a very short period of regular HIIT. Furthermore, in studies that were able to significantly improve the 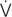
O_2_max of healthy and well-trained athletes, participants usually undertook at least 8 weeks of HIIT (Iaia et al., 2009). A 4-week HIIT program may therefore be too short to effectively increase 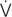
O_2_max in an already well-trained cohort.

While an enhancement in 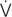
O_2_max with training results primarily from an increase in maximal cardiac output (Bassett and Howley, 2000), the improvement in vmax, v
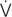
O_2_max, and AT can have multifactorial causes. For example, metabolic adaptations in skeletal muscle are critical for improving submaximal endurance performance (i.e., v
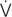
O_2_max, AT; Bassett and Howley, 2000). In this context, it could be shown that several weeks of HIIT led to endurance-like adaptations, including an increased skeletal muscle oxidative capacity, a reduced lactic acid production at a given load, an increased capacity for whole-body and skeletal muscle lipid oxidation, or an enhanced peripheral vascular structure and function (Gibala et al., 2012). Even though we did not measure cellular adaptations, this might partially explain the significant improvements in v
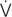
O_2_max and AT observed in this study.

Furthermore, it has been proposed that endurance performance is also influenced by running economy, which in turn is a concept that reflects the integrated composite not only of metabolic and cardiorespiratory capacities (i.e., substrate utilization, 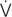
O_2_max) but also of biomechanical and neuromuscular characteristics (i.e., running style, motor programming; Barnes and Kilding, 2015). In this context, Silva et al. (2017) revealed that a 4-week HIIT mesocycle in fact improved running economy. An increase in running economy could lead to a lower energy cost during submaximal running bouts, which might also allow the athletes to achieve higher speeds at the end of the maximal incremental treadmill test (Silva et al., 2017). Taken together, this may explain why the HIIT program performed during 4 weeks was an effective short-term strategy to alter not only submaximal variables related to endurance performance but also vmax.

In contrast to the improvements of vmax, v
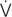
O_2_max, and AT, no changes in RSA (i.e., neither in MV nor in Sdec) were observed. Although ES were small for changes of Sdec in both the whole group, and in the ACT and PAS groups, the magnitude of changes cannot be considered to be of practical relevance due to the insufficient reliability scores of Sdec. These results are in agreement with those of Ferrari Bravo et al. (2008) and Fernandez-Fernandez et al. (2012), who reported an unaltered RSA following a HIIT mesocycle, while RSA was elevated in a group that completed a repeated-sprint training program instead of HIIT. For the sprint group, they found a decrease in the mean time concurrent with an unchanged percentage decrement score during the RSA test. Although RSA may be partially related to aerobic power, this suggests that an improved RSA is primarily due to positive changes in specific coordination and/or overall anaerobic performance rather than the ability to recover between sprints. Consequently, a HIIT program might not be appropriate to optimize the RSA in well-trained intermittent sport athletes.

Besides the knowledge of the effectiveness of HIIT to enhance the fitness of athletes, little is known about the long-term impact of implementing recovery strategies (i.e., ACT) into sport practice on potential training adaptations. Performing low-intensity exercise immediately following HIIT re-establishes the homoeostasis of the cell faster (i.e., accelerated blood lactate removal and/or pH recovery) in comparison with PAS (Wahl et al., 2013). However, accumulated lactic acid is a potent metabolic stimulus, which plays a major role in the control of physiological adaptations and therefore may be essential for improvements in endurance performance (Philp, 2005; Wahl et al., 2010b; Nalbandian and Takeda, 2016). As PAS leads to more long-lasting metabolic alterations (**Figure 2**) and therefore increases the time of exposure to high blood lactate levels, we have hypothesized that PAS during the cooldown phase of each HIIT session would result in greater physiological and performance adaptations than ACT.

In this study, however, we found that ACT did not attenuate but even increased the adaptations to training compared to PAS. Since we did not measure any cellular or molecular mechanisms in response to the 4-week HIIT program, and this is the first study comparing the effects of two different recovery strategies (i.e., ACT and PAS) immediately after HIIT on the chronic physiological and performance responses, the precise reasons for this finding are unclear; however, there are a number of possible explanations. First, the athletes were exposed to higher blood lactate levels during PAS compared to ACT for only a short period of time (**Figure 2**). This timeframe of greater metabolic alterations may be too short for the adaptive potential of lactate to effectively unfold. Second, ACT itself may activate specific adaptive mechanisms that are not triggered during PAS and compensate for the potential negative influence of an accelerated homeostasis normalization on training adaptations. In this regard, it is suggested that low-intensity work is an adequate stimulus to activate signaling pathways and thereby induce the expression of muscle factors (i.e., mitochondrial biogenesis, capillarization, increased oxidative activity) favoring endurance performance (Le Meur and Hausswirth, 2013). Third, Fairchild et al. (2003) have reported that ACT performed immediately after HIIT significantly impaired glycogen synthesis, especially in type I muscle fibers. In this context, it is speculated that prolonged glycogen depletion during training can enhance the adaptive signaling response that is required for mitochondrial biogenesis (Knuiman et al., 2015). Although this is highly speculative, it could be an additional explanation for the superior effect of ACT on submaximal endurance performance (i.e., AT) compared to PAS.

Finally, some limitations of the study design must be considered. First, because we did not have enough time and resources, we could not use a crossover design to investigate the influence of ACT after HIIT on training adaptations, but this would have had some advantages over a parallel design. For example, we would have been able to compare and report individual responses to the two treatments (i.e., ACT and PAS). Second, the HIIT mesocycle induced only a moderate increase in some variables related to endurance performance. Among other things, this may be due to the fact that the training period lasted only 4 weeks, which is probably too short to cause larger adaptations in this well-trained cohort. However, the hypothesized negative influence of ACT on the efficiency of HIIT may only be apparent after longer training periods (i.e., >4 weeks) and/or if larger physiological adaptations are to be expected, but this must remain speculative and warrants further research. Third, due to the multitude of *t*-tests, there is an increased likelihood that the so-called multiple testing problem occurs. That is why we reported ES and made magnitude-based inferences with reference to a smallest worthwhile change to ensure the best possible interpretation of the data. Fourth, we did perform a sample size estimation. Compared to similar studies, however, the number of participants is relatively high. Moreover, we reported CIs, which allow the likely range of the true value to be expressed.

## Conclusion

A 4-week HIIT program with a total of 12 training sessions during the preparatory period (i.e., the off-season) was an effective short-term strategy to alter some variables related to endurance performance in well-trained intermittent sport athletes. Furthermore, the regular use of moderate runs for 15 min (ACT) after HIIT did not attenuate training adaptations—on the contrary, ACT even resulted in a more pronounced increase in the anaerobic threshold compared to PAS without having a beneficial or detrimental effect on chronic HIIT-induced fatigue. As such, maintaining low-intensity work during the cooldown phase of a training session could actually be a means to continue training, thereby activating specific adaptive mechanisms without increasing the state of fatigue, rather than a means to truly accelerate the recovery process. Therefore, a change of habitual post-training cooldown routines seems not to be necessary. However, coaches and athletes should reconsider the actual intention of performing ACT after intensive training.

## Author Contributions

TW, CS, TM, MK, MP, and AF: conceived and designed the experimental design; contributed reagents, materials, and/or analysis tools. TW, CS, AS, and AD: performed the experiments. TW and CS: analyzed the data. TW and AF: wrote the paper.

## Conflict of Interest Statement

The authors declare that the research was conducted in the absence of any commercial or financial relationships that could be construed as a potential conflict of interest.
